# Thermoregulatory responses in elite cross-country skiers during international competitions and training

**DOI:** 10.3389/fphys.2025.1709093

**Published:** 2025-12-11

**Authors:** Wolfgang Schobersberger, Maarit Valtonen, Anika Köck, Sebastien Racinais, Yannis Pitsiladis, Panagiotis Verdoukas, Borja Muniz-Pardos, Rikhard Mäki-Heikkilä, Johanna K. Ihalainen, Dominique Gagnon, Tobias Dünnwald

**Affiliations:** 1 Institute for Sports Medicine, Alpine Medicine and Health Tourism (ISAG), UMIT TIROL-Private University for Health Sciences and Health Technology, Hall in Tyrol, Austria; 2 Tirol Kliniken GmbH Innsbruck, Innsbruck, Austria; 3 Finnish Institute of High Performance Sport KIHU, Jyväskylä, Finland; 4 CREPS Montpellier-Font-Romeu, Environmental Stress Unit, Montpellier, France; 5 Centre for Exercise Science and Medicine (CESAME), Hong Kong Baptist University, Kowloon Tong, Hong Kong SAR, China; 6 Department of Biology, Faculty of Science, Hong Kong Baptist University, Kowloon Tong, Hong Kong SAR, China; 7 Human Telemetrics Ltd., London, United Kingdom; 8 EXER-GENUD (Growth, Exercise, Nutrition and Development) Research Group, Faculty of Health and Sport Sciences, University of Zaragoza, Zaragoza, Spain; 9 Faculty of Medicine and Health Technology, Tampere University, Tampere, Finland; 10 Faculty of Sport and Health Sciences, University of Jyväskylä, Jyväskylä, Finland; 11 Helsinki Clinic for Sport and Exercise Medicine, Helsinki, Finland; 12 Department of Sport and Exercise Medicine, Faculty of Medicine, University of Helsinki, Helsinki, Finland; 13 Center for Research in Occupational Safety and Health, Laurentian University, Sudbury, ON, Canada

**Keywords:** cross-country skiing, competition, thermoregulation, core temperature, skin temperature, cold

## Abstract

**Background:**

The aim of this study was to describe the thermoregulatory responses of elite athletes during competitions and training of the international cross-country skiing FIS Scandinavian Cup in Finland, held under cold (subzero) ambient conditions.

**Methods:**

The core and skin temperatures were continuously recorded during two competition formats—a 10-km race (n = 18; 10 m, 8 f) and a 20-km race (n = 14; 9 m, 5 f)—and during training (n = 11; 7 m, 4 f) using electronic temperature pills and temperature sensors fixed on the chest, arm, hand, and thigh, respectively. The heart rate and skiing speed were continuously recorded using heart rate monitors with integrated GPS technology. Ambient temperatures during the measuring period ranged from −13.0 °C to −1.3 °C.

**Results:**

The mean core temperature (T_core_) increased significantly during the 10-km classic (39.0 °C ± 0.4 °C) and the 20-km freestyle (39.2 °C ± 0.7 °C) races (all p < 0.001) and during trainings (38.3 °C ± 0.5 °C). In contrast, skin temperature decreased in all four body parts (all p < 0.001), with the greatest decreases measured on the thigh [18.7 °C ± 4.1 °C (10-km race), 20.7 °C ± 4.6 °C (20-km race), and 18.5 °C ± 3.2 °C (training)]. During both races, the heart rate significantly increased over time while the racing speed decreased (p < 0.001, respectively). The mean skin temperature of the thigh correlated with skiing speed in the 10-km (r = 0.573, p = 0.041) and the 20-km (r = 0.682, p = 0.021) races.

**Conclusion:**

In summary, despite low ambient temperatures under real competition and training conditions, the athletes exhibited high heat generation, which enabled them to maintain a high core temperature. In contrast, the skin temperature dropped sharply during competitions and trainings. The association between the low mean skin temperature and the lower racing speed should be investigated further.

## 1 Introduction

Cross-country skiing is sometimes performed under adverse weather conditions, with temperatures falling under −15 °C. Currently, data on thermoregulatory responses to the diverse demands of cold-weather sport disciplines are scarce. Importantly, an impaired thermoregulatory response may, in addition to negatively impacting exercise performance (Castellani and Young, 2016), be an indirect risk factor in the epidemiology of injuries and illness in athletes.

Cross-country skiing performance continuously involves the lower and upper body, with sustained metabolic heat production (Holmberg, 2015). Given this high energetic demand, it has been suggested that the risk for hypothermia would be rather low (Bergeron et al., 2012). However, when athletes are unable to maintain thermal homeostasis and when heat loss exceeds heat gain induced by individual metabolic heat production, it may compromise cardiovascular (e.g., lower muscle oxygenation) and neuromuscular function and metabolic regulation (e.g., preference on carbohydrate utilization) (Castellani and Young, 2016), with possible adverse effects on performance and health. Recent observations from a field study performed in national team biathletes showed that the skin temperature (T_skin_) decreased by 7.5 °C (thigh) during submaximal training at −4 °C (Blokker et al., 2022). A decrease in T_skin_ can be relevant, as there is a linear relationship between the impairment of neuromuscular function and the fall in muscle temperature (Bergh and Ekblom, 1979a; Bigland-Ritchie et al., 1992; De Ruiter and De Haan, 2000). It has previously been demonstrated that in highly trained male cross-country skiers, the mean double-poling power output was reduced to a greater extent under simulated cold (−14 °C) than under cool (6 °C) conditions (Wiggen Ø et al., 2013), whereas this was not confirmed in females (Renberg et al., 2014).

The competition rules for cross-country skiing (International Ski and Snowboard Federation, FIS) state that competitions will be postponed or canceled if the temperature falls below −20 °C in the coldest part of the course and that other factors such as the duration of exposure, clothing, and “wind chill factor” have to be considered when a decision is made about starting or continuing a competition (FIS, 2022). During the 2022 Winter Olympic Games held in Beijing, China, very low temperatures combined with high wind speeds led to modifications of certain events, including a delay and shortening of the men’s 50-km cross-country mass start to 30 km, bringing safety and coping issues of winter-sport athletes into broader discussion. The Wind Chill Temperature (WTC) Index can be used to estimate the cooling power of the environment (Lankford and Fox, 2021); however, it does not provide additional data on the individual thermoregulatory responses of the athletes. Although the International Olympic Committee (IOC) already emphasized the assessment of the thermoregulatory responses of elite athletes training in the cold years ago to improve their safety, research in this area is scarce (Bergeron et al., 2012).

Therefore, the aim of this study was to characterize the thermal response of elite cross-country skiers training and competing during an international event in the cold, focusing on the core temperature (T_core_) and T_skin_ alterations.

## 2 Materials and methods

### 2.1 Participants and study design

Thermoregulatory data were collected from 21 elite Finnish cross-country skiers [tier 4 (McKay et al., 2022); FIS Points List- International Ski Federation: DI FIS March 4th, list 20–12–23; mean ± SD: 74 ± 30 (males, m) and 98 ± 38 (females, f)] during two international competitions and one regular training in Vuokatti, Finland.

Competitions were part of the second-level international FIS Scandinavian Cup, which was held in Vuokatti, Finland. On the first competition day, 18 athletes [10 m, 8 f; mean ± SD age: 23 ± 4 years (m), 21 ± 3 years (f); body height: 182 ± 4 cm (m), 170 ± 4 cm (f), p < 0.001; body weight: 79 ± 6 kg (m), 67 ± 4 kg (f), p < 0.001] performed a 10-km race in the classical style. On the second competition day, 14 athletes [9 m, 5 f; age: 25 ± 4 years (m), 22 ± 4 years (f); body height: 182 ± 4 cm (m), 170 ± 5 cm (f), p = 0.001; body weight: 78 ± 6 kg (m), 65 ± 3 kg (f), p < 0.001] completed a 20-km freestyle race. A total of 13 athletes took part in both competitions (8 m, 5 f). T_core_ and T_skin_ during training were measured in 11 athletes [7 m, 4 f; age: 24 ± 6 years (m), 19 ± 2 years (f); body height: 180 ± 6 cm (m), 169 ± 4 cm (f) p = 0.030; body weight: 78 ± 7 kg (m), 66 ± 3 kg (f), p = 0.011] over a period of 2 days prior to the competitions.

### 2.2 Competition and training characteristics

During the races, the athletes skied in a ∼3.3 km course (i.e., three and six laps in the 10-km and 20-km races, respectively). The duration of warm-up was 39.2 ± 9.5 min and 34.1 ± 9.0 min on the 10-km and 20-km race days, respectively. On the training days, the course was selected on an individual basis. Training sessions started with an outdoor 33 ± 7 min warm-up skiing session. The main part of training lasted 35 ± 3 min on average and consisted of several longer intervals (10 min–12 min) at intensities corresponding to ∼70%–80% of the individual maximum heart rate, each interspersed by 2 min–3 min of active recovery, or was performed continuously.

### 2.3 Environmental conditions and ski track

Data on the environmental conditions including average ambient temperature (°C), wind speed (km·h^−1^), and relative humidity (%) were obtained from a nearby weather station (Table 1). The starting point of the race was located at an altitude of 150 m, with a maximum elevation reaching 178 m above sea level. The racetrack consisted of several uphill (total distance (td): ∼1,350 m), downhill (td: ∼1,350 m), and flat (td: ∼750 m) sections.

**TABLE 1:** Lowest and highest T_core_, T_core_-to-T_skin_ gradient, and environmental conditions.

Exercise condition	Lowest T_core_ (minimum) [°C]	Peak T_core_ (maximum) [°C]	T_core_-to-T_skin_ gradient (last lap) [°C]	T_core_-to-T_skin_ gradient (all laps) [°C]	Ambient temperature [°C]	Wind speed [km·h^−1^]	Relative humidity [%]
Race_10 km_ (m)	37.8 ± 0.6 (37.2)	39.2 ± 0.4 (39.9)	14.6 ± 1.3	14.2 ± 1.0	−6	11	95
Race_10 km_ (f)	37.8 ± 0.5 (36.7)	39.1 ± 0.5 (39.9)	18.0 ± 1.4**	17.5 ± 1.2**	−7	7	94
Race_20 km_ (m)	38.0 ± 0.3 (37.5)	39.3 ± 0.6 (39.9)	16.0 ± 1.8	15.4 ± 1.9	−2	20	88
Race_20 km_ (f)	37.8 ± 0.6 (37.1)	39.0 ± 1.1 (40.0)	20.6 ± 2.2*	19.8 ± 1.4*	−1	18	88
Training (m)	37.7 ± 0.4 (37.1)	38.5 ± 0.4 (38.9)	-	-	−13	11	92
Training (f)	37.7 ± 0.3 (37.3)	38.5 ± 0.4 (38.9)	-	-	−13	11	92

Data are presented as the means ± SD (minimum/maximum). (m) males and (f) females. *p ≤ 0.01 and **p ≤ 0.001 for significant differences compared to males. T_core_, core temperature. T_skin_, skin temperature. 10-km race (three laps), 20-km race (six laps).

### 2.4 Measurements

#### 2.4.1 Core and skin temperature

T_core_ was recorded via the electronic (gastro-intestinal) temperature pill at 60-s intervals with a precision of 0.1 °C (eCelsius capsule BodyCap, Caen, France). The pill was ingested by the athletes 4 h–6 h before the start of the training/race. Data were transmitted via low-bandwidth radio frequency to a data logger (ePerf Connect Gateway, BodyCap, Caen, France) carried by the athletes during races and trainings. T_skin_ was continuously measured with a flexible thermistor (eCelsius flex; BodyCap, Caen, France) placed on the chest, forearm, thigh, and back of the hand. The reference baseline for T_core_ and T_skin_ was calculated as an average of a 5-min rest in a temperate room (∼22 °C) before warm-up. For each lap, the mean T_core_ and mean T̅_skin_ (regular average of all four locations) were used to calculate the T_core_-to-T_skin_ gradient.

#### 2.4.2 Heart rate and speed

Heart rate (HR) and speed were continuously recorded using heart rate monitors equipped with GPS (Polar Vantage V3 with Polar H9 chest strap, Polar Electro, Kempele, Finland; Forerunner 35, with HRM-Pro, Garmin, Olathe, KS, United States).

#### 2.4.3 Body mass and sweat loss

Body mass (Detecto, Knorring, Finland) was measured prior to warm-up and immediately after the cool-down period. Sweat loss was indirectly estimated based on body mass changes corrected for food and fluid intake [by weighing food (energy bars/gels/fruits) and beverages (water/sport drinks)] and for urine output (i.e., 0.3L/pee).

#### 2.4.4 Perceptual measures

Thermal sensation and thermal comfort were assessed using a visual color scale ranging from blue (very cold, 0) to red (very hot, 20) and from white (comfortable, 0) to black (very uncomfortable, 20), respectively. Scores were displayed on the back side of the visual scales and were visible only to the research worker. The 15-point BORG scale, ranging from 6 (no exertion at all) to 20 (extremely hard), was used to evaluate the individual subjective level of perceived exertion (Borg, 1982). Thermal sensation, thermal comfort, and perceived exertion were all determined immediately following competitions or training.

#### 2.4.5 Clothing

The outer layer on both the race days and training consisted of a regular racing skin-suit. The clothing on the day of the 10-km race consisted of a thermal base layer, which for 67% of the athletes consisted of a long-sleeved shirt and long base pants, whereas the remaining athletes wore a short-sleeved base layer shirt and short or no base pants. For the 20-km race, athletes wore short base pants (36%) or only underwear (64%), with one athlete using long base pants. On the upper body, 27% of the athletes wore a long shirt, no shirt (27%), or a T-shirt (46%). Moreover, athletes in the 10- and 20-km races wore a headband (42% and 64%) or a beanie hat (58% and 36%), a scarf (25% and 9%), and thin racing gloves (75% and 91%). During warm-up, athletes wore additional warm-up pants and a jacket. During training, the athletes’ clothing consisted of long thermal undergarments including a long shirt and long pants. In addition, they were equipped with thick beanies and thick race gloves.

### 2.5 Statistical analysis

A Shapiro–Wilk test was applied to test for normal distribution of data. T_core_ and T_skin_ data were averaged for each lap for racing and in 10 segments for training. In addition, the mean T̅_skin_ was calculated as a regular average of the data obtained from all four skin sensor locations. A repeated measures analysis of variance (ANOVA) was then performed to assess changes over time in T_core_ and T_skin_, HR, and speed. Bonferroni-corrected *post hoc* comparisons were conducted in case of significant main effects. Changes in bodyweight (pre- to post-competition/training) were examined using a dependent samples t-test. For those athletes who took part in both races (n = 13), a 2 × 3 (setting x time) repeated measures ANOVA was used to compare changes in T_core_ and T_skin_ from lap 1 to lap 3 of the 10-km and 20-km races. In addition, to compare the perceived exertion, thermal sensation, and thermal comfort (10-km vs. 20-km races) in these athletes, dependent samples t-tests were carried out. Differences between males and females in perceived exertion, thermal sensation, and thermal comfort were assessed using independent samples t-tests. The Pearson correlation coefficient was used to assess correlations between individual variables. Data are presented as the means ± standard deviation (M±SD). The statistical significance was accepted at p ≤ 0.05. All calculations were performed using the IBM SPSS statistical software package for Windows (version 29.0; IBM Corporation, Armonk, NY, United States).

## 3 Results

### 3.1 Core temperature alterations during the competition

**FIGURE 1:**
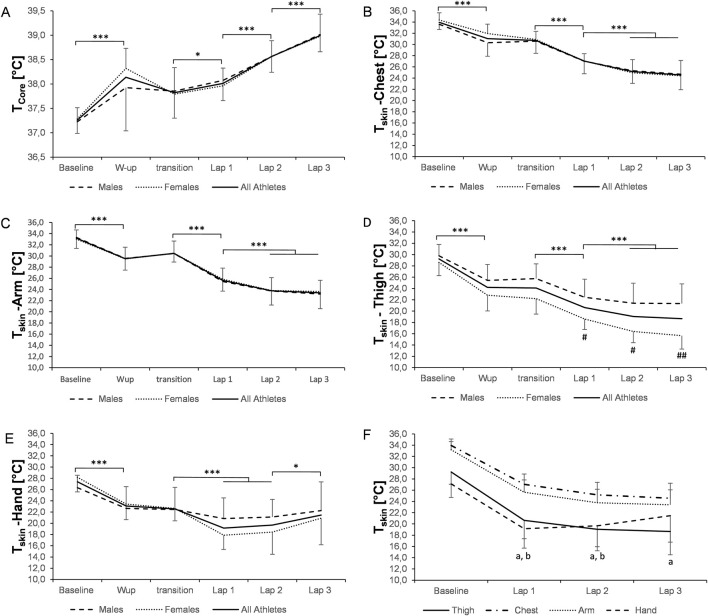
Changes in core [**(A)**, n = 13) and skin **(B–F)**, chest (n = 18), arm (n = 16), thigh (n = 17), and hand (n = 13) **(E,F)**] temperature during the 10-km race. Data are presented as the means ± SD. *p ≤ 0.05, **p ≤ 0.01, and ***p ≤ 0.001 for significant differences. ^a^significantly lower thigh temperature than chest (lap 1–3: p ≤ 0.001) and arm (lap 1–2: p ≤ 0.001; lap 3: p ≤ 0.01) temperatures. ^b^significantly lower hand temperature than the chest (laps 1–2: p ≤ 0.001) and arm (lap 1: p ≤ 0.001; lap 2: p ≤ 0.01) temperatures, respectively. ^#^p ≤ 0.05 and ^##^p ≤ 0.001 significantly different between males and females. A p-value ≤0.05 was considered statistically significant. T_core_, core temperature. T_skin_, skin temperature.

**FIGURE 2:**
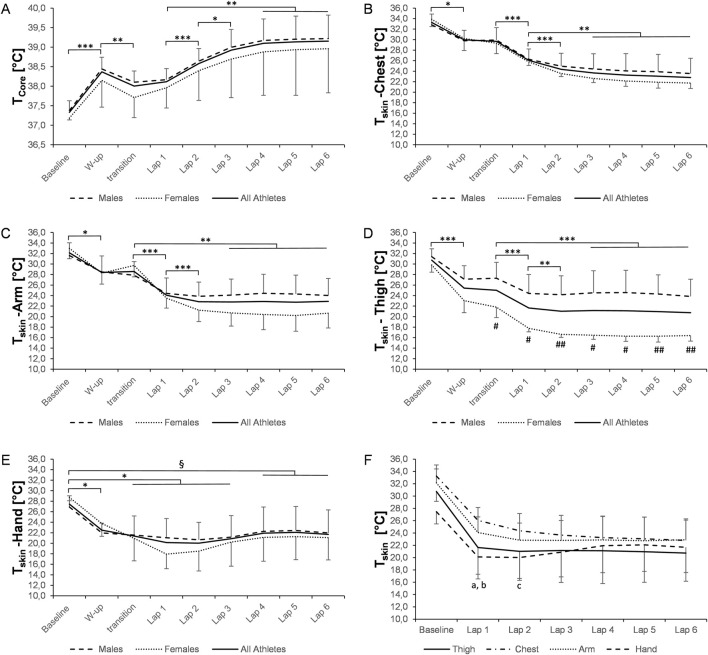
Changes in core [**(A)**, n = 12) and skin **(B–F)**, chest (n = 12), arm (n = 13), thigh (n = 12), and hand (n = 10)] temperature during the 20-km race. Data are presented as the means ± SD. *p ≤ 0.05, **p ≤ 0.01, and ***p ≤ 0.001 for significant differences. ^a^significantly lower temperature on the thigh versus the chest (lap 1: p ≤ 0.01). ^b^significantly lower hand temperature than chest (lap 1: p ≤ 0.001; lap 2: p ≤ 0.05) and arm (lap 1: p ≤ 0.05) temperatures. ^#^p ≤ 0.05 and ^##^p ≤ 0.001 significantly different between males and females. A p-value ≤0.05 was considered statistically significant. T_core_, core temperature. T_skin_, skin temperature.

**FIGURE 3:**
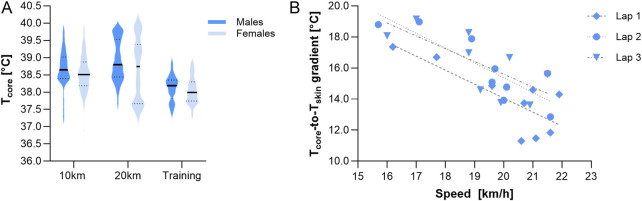
**(A)** Violin plots of core temperatures during the 10-km race (n = 13), 20-km race (n = 12), and training (n = 11), including the median (bolt line) and quartiles (dotted lines). **(B)** Relationship between the T_core_-to-T_skin_ gradient and skiing speed in laps 1 to 3 of the 10-km race. T_core_, core temperature. T_skin_, skin temperature.

The race T_core_ significantly increased over time during the races (p < 0.001, respectively) (Figures 1A, 2A). There was no interaction effect when changes in T_core_ from the first to the third lap were compared between the same athletes taking part in the 10-km and 20-km races (p = 0.263). T_core_ increased until lap 3 in both races and remained elevated during the second half of the 20-km race (Figure 2A). The violin plot in Figure 3A illustrates the distribution of T_core_ recordings during the races. There were no significant differences in the lowest and peak race T_core_ (Table 1).

### 3.2 Skin temperature alterations during competition

There was a significant time effect for the mean T̅_skin_ in the races (both p < 0.001), with a decrease from 31.2 °C ± 1.5 °C to 22.1 °C ± 2.1 °C and from 31.1 °C ± 1.5 °C to 21.9 °C ± 2.7 °C during the 10-km and 20-km races (p < 0.001, respectively, Figures 1, 2). In addition, the mean T̅_skin_ was significantly lower in the 20-km race than that in the 10-km race (22.3 °C ± 2.7 °C vs. 23.9 °C ± 2.1 °C, respectively; p = 0.010). There was no interaction effect for changes in T_skin_ from the first to the third lap between the 10-km and 20-km races (p = 0.227, p = 0.655, p = 0.445, and p = 0.179 for T_skin_ of the thigh, chest, arm, and hand, respectively).

The temperature on each body part significantly changed over time (p < 0.001, respectively). In detail, in both the races, T_skin_ of each location initially decreased during warm-up (p ≤ 0.05, respectively; Figures 1B–E, 2B–E) and further dropped during the first lap (all p ≤ 0.05), except T_skin_ of the hand (20 km), showing no further reduction after warm-up. T_skin_ of the thigh, chest, and arm continued to fall in lap 2 of both the races (all p ≤ 0.05) while remaining constant during the rest of the race.

The lowest individual T_skin_ during the 10-km race was as follows: 12.7 °C (thigh, lap 3), 17.0 °C (chest, lap 3), 18.0 °C (arm, lap 3), and 14.3 °C (hand, lap 2). During the 20-km race, the lowest T_skin_ recorded was as follows: 14.4 °C (thigh, lap 6), 14.9 °C (chest, lap 6), 16.9 °C (arm, lap 6), and 14.9 °C (hand, lap 2). T_skin_ of the thigh was significantly lower during both races in female athletes than in male athletes (all p ≤ 0.05; Figures 1, 2). Differences in T_skin_ between the body parts are presented in Figures 1F, 2F for the 10-km and 20-km races, respectively.

The T_core_-to-T_skin_ gradient was higher in females than in males in the 10-km race (p ≤ 0.001) and the 20-km race (p < 0.010) (Table 1). No correlations were observed between T_core_ and the racing speed. However, the T_core_-to-T_skin_ gradient of each lap was inversely related to the racing speed in laps 1 to 3 of the 10-km race (−0.788, p = 0.012; −0.856, p = 0.003; and −0.723, p = 0.028, respectively, Figure 3B) and tended to be related to the speed in laps 1 and 2 of the 20-km race (−0.570, p = 0.085 and −0.585, p = 0.076, respectively).

### 3.3 Core and skin temperature alterations during training

**FIGURE 4:**
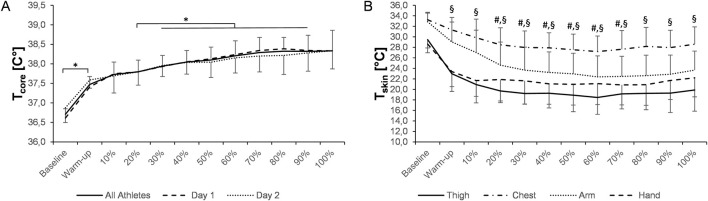
Changes in core [**(A)**, n = 13] and skin (chest, arm, thigh, and hand; **(B)**, n = 5) temperature during training. Data are presented as the means ± SD. T_core_: *p ≤ 0.05 and ***p ≤ 0.001 for significant differences. T_skin_: *for significant differences in T_skin_ of the thigh versus baseline and versus warm-up (p ≤ 0.05, respectively). ^#,§^ denotes significant changes compared to baseline for temperatures measured on the chest (p ≤ 0.05) and arm (p ≤ 0.01), respectively. ^‡^for trends toward lower T_skin_ of the hand versus baseline (p ≤ 0.10). A p-value ≤0.05 was considered statistically significant. T_core_, core temperature. T_skin_, skin temperature.

During training, there was a significant effect of time on T_core_ (p < 0.001), showing a first significant increase during warm-up (p < 0.001) and a second one between the 20% and 30% segment (p ≤ 0.05; Figure 4A). The peak individual T_core_ were 38.9 °C in both the sexes. The lowest T_core_ was 37.1 °C in males and 37.3 °C in females, which were recorded at the beginning of the main training part after warm-up. The mean T_core_ during the end of training (38.5 °C ± 0.5 °C; 100% segment) was significantly lower than that in lap 3 (39.2 °C ± 0.4 °C) and lap 6 (39.4 °C ± 0.5 °C) of the 10-km and 20-km race (p = 0.039 and p < 0.001, respectively).

A significant time effect for the mean T̅_skin_ (all locations) was detected (p < 0.001), declining from 31.4 °C ± 1.4 °C (baseline) to 23.7 °C ± 1.5 °C (100% segment). The mean T̅_skin_ did not differ between females (23.8 °C ± 1.2 °C) and males (23.1 °C ± 1.7 °C; p = 0.55). In addition, temperatures on the thigh, chest, arm, and hand significantly changed over time (p < 0.001, respectively, Figure 4B). Comparisons between the body parts revealed significant differences in T_skin_ depending on the measured location (Figure 4B). The lowest individual T_skin_ during training was as follows: 12.6 °C (thigh), 21.5 °C (chest), 18.4 °C (arm), and 15.1 °C (hand).

### 3.4 Heart rate and speed during the competition and training

**FIGURE 5:**
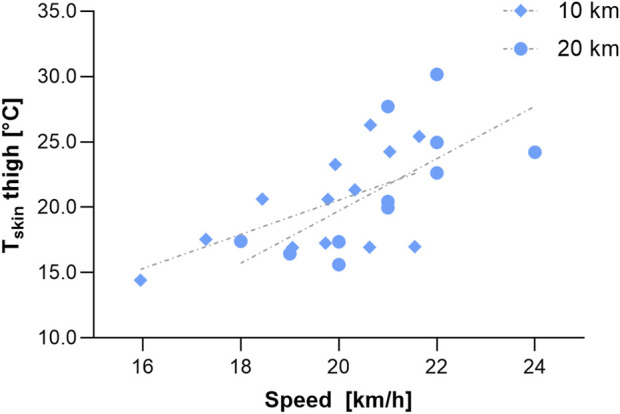
Relationship between the mean T_skin_ of the thigh and skiing speed in the 10-km and the 20-km race.

Alterations in the competition HR and speed are presented in Table 2. In both races, there was a significant effect of time on the HR (increase) and speed (decrease; p < 0.001, respectively). The magnitudes of the changes over time in the HR and speed were not significantly different between males and females during the 10-km race (p = 0.555 and p = 0.875, respectively) and the 20-km race (p = 0.209 and p = 0.429, respectively). However, the speed was significantly lower in females than in males in each lap of the two races. The mean T_skin_ of the thigh was significantly related to the racing speed in the 10-km race (r = 0.573, p = 0.041) and the 20-km race (r = 0.682, p = 0.021) (Figure 5). In addition, the change in T_skin_ of the thigh (baseline to lap 6; 20-km race) was negatively related to the mean racing speed (r = −0.749, p = 0.008).

**TABLE 2:** Average changes of heart rate and speed during the 10-km and 20-km races.

Measured variable	Lap 1	Lap 2	Lap 3	Lap 4	Lap 5	Lap 6	ANOVA P_time_(η_p_^2^)	
HR_10_ [bpm] (m + f)	175 ± 7	181 ± 6***	182 ± 6***^a^				<0.001	(0.826)
*HR*_*10*_ *[bpm] (m)*	175 ± 8	181 ± 7***	183 ± 7***					
*HR*_*10*_ *[bpm] (f)*	175 ± 3	181 ± 4***	181 ± 2**					
Speed_10_ [km/h] (m + f)	20.4 ± 1.8	19.7 ± 1.8**	19.5 ± 1.7**				<0.001	(0.570)
*Speed*_*10*_ *[km/h] (m)*	21.4 ± 0.7	20.7 ± 1.0*	20.5 ± 0.9**					
*Speed*_*10*_ *[km/h] (f)*	18.6 ± 1.7^b^	18.0 ± 1.6^b^	17.7 ± 1.2*^b^					
HR_20_ [bpm] (m + f)	172 ± 7	179 ± 6***	179 ± 7***	178 ± 6**	177 ± 6	176 ± 5	<0.001	(0.632)
*HR*_*20*_ *[bpm] (m)*	170 ± 8	178 ± 7***	178 ± 8***	177 ± 7**	176 ± 7*	177 ± 6**		
*HR*_*20*_ *[bpm] (f)*	176 ± 5	181 ± 3**	182 ± 3*	180 ± 2	178 ± 2	178 ± 2		
Speed_20_ [km/h] (m + f)	22.2 ± 1.4	21.8 ± 1.6	21.0 ± 1.5***^a^	20.4 ± 1.5***	20.2 ± 1.5***	20.4 ± 1.6***	<0.001	(0.763)
*Speed*_*20*_ *[km/h] (m)*	23.0 ± 0.7	22.7 ± 0.7	21.7 ± 1.1**^a^	21.1 ± 1.2***	20.9 ± 1.2***	21.2 ± 1.1***		
*Speed*_*20*_ *[km/h] (f)*	20.5 ± 0.6^b^	19.7 ± 1.0^b^	19.3 ± 0.9^b^	18.9 ± 0.5*^b^	18.6 ± 0.6**^b^	18.7 ± 1.2*^b^		

Data are presented as the means ± SD. 10-km race (n = 14; 9 m, 5f; three laps), 20-km race (n = 13; 9 m, 4f; six laps). Significantly different compared to lap 1 *p ≤ 0.05, **p ≤ 0.01, and ***p ≤ 0.001.

^a^ significantly different compared to the previous lap (p ≤ 0.05).

^b^ significantly different between males and females (p ≤ 0.01). η_p_2 = partial eta squared. ANOVA, analysis of variance; HR, heart rate. (m) males and (f) females.

During training, the mean HR was 166 ± 6 bpm and 133 ± 8 bpm for the intense and active recovery parts, respectively. The corresponding skiing speeds were 18 ± 3 km/h and 3 ± 3 km/h.

### 3.5 Body weight, nutritional intake, and estimated water loss (during the competition and training)

Body weight significantly decreased from pre- to post-skiing during the 10-km race (73.3 ± 8.0 kg to 72.7 ± 8.1 kg; −0.9% ± 0.7%, p < 0.001) and the 20-km race (73.3 ± 8.2 kg to 72.5 ± 8.1 kg; −1.0% ± 0.7%, p < 0.001). During training, body weight changes were not significant (from 72.8 ± 7.8 kg to 72.5 ± 7.4 kg, −0.4% ± 0.7%, p = 0.104). The mean fluid and food intake was 0.8 ± 0.4 kg (10-km race), 0.6 ± 0.3 kg (20-km race), and 0.4 ± 0.2 kg (training). Considering urine output, the estimated sweat loss was 0.8 ± 0.5 L (10-km race), 0.8 ± 0.6 L (20-km race), and 0.6 ± 0.6 L (training).

No effect of sex on changes in body weight over time were observed (p = 0.553). Body weight loss in males and females was −0.5 ± 0.3 kg and −0.8 ± 0.5 kg in the 10-km race and −0.7 ± 0.5 kg and −0.8 ± 0.6 kg in the 20-km race, respectively. Similarly, the estimated sweat loss did not differ between males and females during the 10-km race (−0.8 ± 0.5 L and −0.8 ± 0.5 L, respectively; p = 0.839) and 20-km race (−0.8 ± 0.4 L and −0.8 ± 0.9 L, respectively; p = 0.917).

Body weight changes during training were significantly different between males and females (−0.56 ± 0.46 kg and 0.18 ± 0.31 kg, p = 0.020). In addition, sweat loss was higher in males than in females (−0.84 ± 0.59 L and −0.10 ± 0.33 L, p = 0.048, respectively).

### 3.6 Perceived exertion and subjective thermal sensation (competition and training)

The mean scores of the Borg scale were 18 ± 2 (10-km race), 17 ± 2 (20-km race), and 14 ± 1 (training). For athletes taking part in both races, the scores tended to be higher in the 10-km race than in the 20-km race (18.0 ± 1.6 and 16.9 ± 2.1, respectively; p = 0.061). Scores were not different between males and females during the 10-km race (17.7 ± 1.6 and 17.6 ± 1.8, respectively; p = 0.976) and the 20-km race (16.8 ± 2.4 and. 17.2 ± 1.5, respectively; p = 0.719).

The athletes’ thermal sensation and thermal comfort was 9.3 ± 3.3 and 6.6 ± 3.8 (10-km race), 10.5 ± 3.8 and 4.9 ± 2.6 (20-km race), and 6.3 ± 2.5 and 8.1 ± 4.4 (training), respectively. Ratings for thermal sensation and thermal comfort in athletes that were examined on both the racing days did not differ (p = 0.21 and p = 0.30, respectively). Thermal sensation was significantly higher during the 10-km race in males than in females (10.9 ± 3.4 vs. 7.3 ± 2.2; p = 0.02) and tended to be higher during the 20-km race (12.3 ± 3.0 vs. 8.1 ± 4.4; p = 0.07). Thermal comfort was not different between males and females in the 10-km and 20-km races (6.3 ± 3.7 vs. 6.9 ± 4.4; p = 0.77 and 4.4 ± 2.7 vs. 5.2 ± 3.0; p = 0.64, respectively).

Thermal comfort was related to changes in T_core_ (r = 0.619, p = 0.024) in the 10-km race. In addition, thermal sensation and thermal comfort tended to be negatively related in the 10-km (r = −0.424, p = 0.080) and the 20-km races (r = −0.516, p = 0.059), respectively.

## 4 Discussion

The aim of the present study was to examine and describe the thermoregulatory response of elite cross-country skiers during international FIS competitions and preparatory trainings. Our findings provide novel insights into the changes of T_core_ and T_skin_ during cross-country skiing competitions performed under cold (sub-zero) environmental conditions, showing that the mean T_core_ increased to ≥39 °C during the shorter (10-km race) and the longer (20-km race) distance of the race. Conversely, we observed a considerable decline in skin temperatures at all measured locations.

### 4.1 Core temperature

There were no athletes with an individual T_core_ below the lower cut-off for normal core temperature during the races (i.e., all >36.5 °C, Table 1). Therefore, our results corroborate previous assumptions made for Nordic skiing sports, that is, that metabolic heat production is high and sufficient to compensate for body heat loss under the given environmental, clothing, and race conditions (Bergeron et al., 2012).

We observed a fast reduction in T_core_ between the warm-up and the start of the race on the day of the 10-km (−0.4 °C; Figure 1A) and 20-km (−0.3 °C; Figure 2A) races, highlighting the benefit of an appropriate heating strategy to maintain the body temperature during transition periods. This may be of particular relevance, as recent research suggested that such strategies may not be part of the planning of warm-up routines in cross-country skiers (Jones et al., 2022).

The marked increase in T_core_ during the race can be explained by the high metabolic demands of the skiing activity, requiring intensive energy expenditure that simultaneously involves muscles of the upper and the lower body (Holmberg, 2015). The hilly terrain poses an additional stressor on the athlete, especially when steep uphill sections have to be managed (Norman et al., 1989). This can be relevant, as generally half of the total race time during comparable competitions is spent in uphill sections, where work rates can clearly exceed 100% of V̇O_2peak_ (Losnegard, 2019). Previous laboratory studies performed in cross-country skiers showed that T_core_ significantly increased (>38.0 °C) during 20 min of maximum double-poling exercise at −15 °C (Wiggen Ø et al., 2016) and that submaximal running (−9 °C, wind speed 5 m·s^−1^) raised T_core_ up to 38.5 °C in athletes wearing cross-country skiing racing skin-suits (Sandsund et al., 2012; Renberg et al., 2014). In our study, we add novel knowledge by considering a wider range of factors impacting on the thermoregulatory response, that is, including real outdoor weather conditions combined with specific demands of the competition setting, such as variations in terrain and movement velocity, which are partially responsible for the larger increases in T_core_ in our study. Notably, race T_core_ recorded in our study significantly exceeded that reported from alpine skiing (37.6 °C) (Alhammoud et al., 2021). Despite the subzero outdoor temperatures, athletes’ T_core_ continuously increased until ∼39 °C during the third lap and was maintained throughout the remaining three laps of the 20-km race. By reaching such levels, the cross-country skiers of our study represented values comparable to the mean peak T_core_ (39.2 °C) shown in road-racing athletes during the UCI Road Cycling World Championships (Racinais et al., 2019) or running events performed in temperate/hot conditions (Grivas et al., 2024). Thus, even under the cold conditions prevailing in our study, the workload of exercise appears to be a more discriminating factor regulating T_core_ than the environment the exercise is performed in.

Our finding that T_core_ is not associated with the racing speed is consistent with previous observations made during competitions in the heat (Aylwin et al., 2023), suggesting that factors other than T_core_ may also be responsible for the decline in performance when training or competing in a cold environment. Notably, we found that a higher T_core_-to-T_skin_ gradient was related to a reduced racing performance (Figure 3B). Under hot conditions, a reduced T_core_-to-T_skin_ gradient has been suggested as a determining factor negatively affecting the exercise capacity during competitions in elite athletes (Racinais et al., 2022). In addition, the T_core_-to-T_skin_ gradient was found to better reflect fatigue than T_core_ in the heat (Cuddy et al., 2014). The lower gradient may be associated with increased skin blood flow, thus diminishing available blood for working muscles, with possible consequences such as reduced oxygen delivery to muscles or higher cardiac strain. Although a high gradient under hot conditions may be associated with better heat tolerance and exercise capacity, a large T_core_-to-T_skin_ gradient during exercise in the cold may be an indirect indicator of performance degradation. A low skin temperature may result in the cooling of superficial muscles, possibly impairing enzymatic and neural processes (Drinkwater, 2008; Racinais and Oksa, 2010), which are factors that may limit skiing performance. Nevertheless, it is important to consider that our findings do not imply causality.

During training, outdoor temperatures and exercise intensities were considerably lower than those during racing days, yet T_core_ increased to 38.3 °C on average (Figure 4A). The magnitude of the T_core_ increase despite the colder ambient temperatures combined with lower skiing speeds can be partly explained by the additional clothing worn during the training sessions. Our findings are in line with previous observations, reporting a rise in T_core_ during on-snow training in biathletes at comparable exercise intensities (14 min, 78% of HR_max_), although changes in T_core_ were less pronounced (i.e., from 37.0 °C to 37.5 °C) albeit markedly warmer ambient temperatures (−3.7 °C) (Blokker et al., 2022).

### 4.2 Skin temperature

Conversely to higher T_core_, skin temperatures declined on all four body parts during races and training, particularly affecting the distal regions (Figures 1B–E, 2B–E, 4). Despite quadriceps musculature being one of the most involved muscles to generate propulsive forces during cross-country skiing movements, we observed large decreases in T_skin_ measured on the thigh. These results confirm the findings from earlier studies performed under cold conditions in the lab, reporting distinct falls in skin temperature, especially of the thigh (Sandsund et al., 2012; Wiggen Ø et al., 2013; Wiggen Ø et al., 2016). Under milder but real outdoor conditions, T_skin_ of the thigh was again the most affected, showing a drop down to ∼23 °C (Blokker et al., 2022). Interestingly, the mean T_skin_ and temperature of the thigh were much lower than the average values previously reported during alpine skiing training (30.5 °C and 29.3 °C, respectively). The underlying mechanism for the decrease in T_skin_ can be manifold. For example, peripheral vasoconstriction together with a decrease in skin blood flow may diminish convective heat loss as an acute response to cold exposure (Castellani and Young, 2016). Conversely, the sharp reduction in T_skin_ observed in our study may also stem from high evaporative heat loss. Although outdoor temperatures were in the subzero range, athletes substantially lost body mass (equivalent to a loss of approximately 1% of athletes’ body weight), with evaporative heat loss likely accounting for the most part of this observed change.

We observed some interindividual variability, with faster athletes exhibiting greater average temperatures on their thighs during the races. In the 20-km race, lower mean racing speed was associated with greater decreases in T_skin_ of the thigh. However, due to the observational nature of the study design, it is not possible to determine if the lower speed was a cause for or consequence of the lower T_skin_. Thus, this finding may simply reflect greater muscular heat production because of a higher workload. Yet, we cannot exclude that lower muscle temperature might have limited force production and, thus, impaired performance. A lower skin temperature may be associated with subcutaneous muscle tissue cooling (Oksa et al., 1995; Racinais and Oksa, 2010) and/or can prevent muscle temperature to increase to similar levels as under thermoneutral conditions (Bergh and Ekblom, 1979b). Cooling of the superficial muscles was suggested to appear in a dose-dependent manner (Bortolan et al., 2021), with mild cooling already inducing considerable performance impairments (Oksa et al., 1997). For example, cooling the leg muscles down to 29 °C led to a 38% reduction in time to exhaustion in cycling exercise, rather than when the muscles were at warmer temperatures (e.g., to 34 °C) (Blomstrand et al., 1984). In this regard, dynamic exercise performance was suggested to be more sensitive to variations in muscle temperature than isometric contractions (Wakabayashi et al., 2015).

As the potential underlying mechanism for reduced performance, impairments in neuromuscular function (e.g., slowing down of nerve conduction and muscular contraction velocity), increased co-activation (alteration in the agonist-antagonist relation), changes in the muscle metabolism (reduced blood flow and oxygen uptake), and/or decreases in exercise economy/efficiency have been postulated (Racinais and Oksa, 2010; Wakabayashi et al., 2015; Castellani and Young, 2016). In elite alpine skiers, cold-induced impairments in proprioceptive acuity (i.e., pattern of error) were reported following exposure to cold, whereas cognitive task performance remained unchanged, possibly due to cold habituation (Racinais et al., 2017). Decreased muscular functioning may overload the musculoskeletal system due to the known high demands of cross-country skiing, possibly increasing the risk for muscular disorders or injuries, although evidence underpinning this assumption is still lacking. Cold-induced increase in tissue stiffness and impaired proprioception may be factors affecting the injury risk of athletes (Mäkinen et al., 2005; Racinais and Oksa, 2010; Lauersen et al., 2014).

### 4.3 Differences between sexes

After the start of the race, T_core_ (Figures 1A, 2A), representing heat storage, and HR (Table 2) increased to a similar extent in males and females, however for a lower absolute racing speed in females (Table 2). Due to generally lower body mass, power output in females is lower, which would result in less thermal energy production for the same speed. At the same time, the body surface area is smaller in females, implying less heat dissipation capacity (Notley et al., 2017; Notley et al., 2021). Due to lower body weight, and as thermal energy stored for each °C is proportional to the body mass, females appear to have stored less heat than males.

Interestingly, Renberg et al. (2014) reported no effects of simulated cold temperatures (−14 °C) in females on various performance parameters (e.g., running speed/economy), even though T_skin_ was significantly lower than that in males under similar conditions (Sandsund et al., 2012). Authors suggested females being able to tolerate lower skin temperatures, possibly due to greater subcutaneous fat thickness, that may have prevented their muscle temperature from falling (Renberg et al., 2014). In addition, skin temperature was shown to decrease more when fat thickness increased (Castellani and Tipton, 2015). Likewise, in our study, T_skin_ of females was affected more by temperature than that of males. Yet, changes in the racing performance were not significantly different between sexes, as indicated by similar reductions in racing speed (i.e., −0.9 km/h and −1.8 km/h in the 10-km and 20-km races, respectively).

### 4.4 Thermal sensation and thermal comfort

The significantly lower T_skin_ of the thigh in females may explain their lower thermal-sensation scores than those of males. However, although T_skin_ considerably declined in both males and females (particularly on the thigh), this decrease does not seem to be adequately reflected in the ratings of thermal sensation and thermal comfort. This suggests a perception closer to a neutral temperature rather than a true feeling of cold, accompanied by low levels of thermal discomfort. Therefore, there may be a general disparity between temperatures measured on the skin and the athlete’s subjective thermal sensation/comfort on the days of the races. These findings corroborate with previous observations, indicating depressed thermal sensation during exercise in the cold (Ouzzahra et al., 2012; Blokker et al., 2022). As both T_core_ and T_skin_ are known to equally contribute to the sensation of thermal comfort, a factor that is crucial for behavioral thermoeffector initiation (Frank et al., 1999), it is unlikely that the observed discrepancy would be solely based on the high T_core_ observed in our study. Nevertheless, relative differences in outdoor temperatures appear to have been adequately sensed by the athletes, showing higher ratings for thermal sensation and lower ratings for thermal comfort on warmer days.

## 5 Limitations

We were not able to analyze T_core_ data from all the ingested pills. Due to technical issues during data recordings/transfer, five (4 m and 1 f) out of 18 datasets in the 10-km race and two (2 f) out of 14 in the 20-km race could not be retrieved. In addition, we were not able to record the skin temperature data on all locations during races and training (see figure legends).

## 6 Conclusion

In this study, cross-country skiers produced high amounts of body heat, as indicated by large increases in T_core_ during real competitions and training in the cold. At the same time, however, T_skin_ markedly dropped across the races and during training, which can be problematic, as reductions in T_skin_ may facilitate superficial muscle cooling. It remains to be established how thermoregulation and performance are affected during longer race distances and/or during more adverse conditions, and if T_skin_ together with the T_core_-to-T_skin_ gradient could be used as suitable indicators for exercise performance in the cold, even more than core temperature alone.

## Data Availability

The raw data supporting the conclusions of this article will be made available by the authors, without undue reservation.
