# Correction: Thermoregulatory responses in elite cross-country skiers during international competitions and training

**DOI:** 10.3389/fphys.2025.1768264

**Published:** 2026-01-09

**Authors:** Frontiers Editorial Office

**Affiliations:** Frontiers Media SA, Lausanne, Switzerland

**Keywords:** cross-country skiing, competition, thermoregulation, core temperature, skin temperature, cold

Errors were made in the affiliation list for author **Yannis Pitsiladis**.

Affiliation ‘Centre for Exercise Science and Medicine (CESAME), Hong Kong Baptist University, Kowloon Tong, Hong Kong SAR, China’ was omitted for author Yannis Pitsiladis. This affiliation has now been added for author Yannis Pitsiladis.

Author Yannis Pitsiladis was erroneously assigned to affiliations ‘Faculty of Science, Hong Kong Baptist University, Kowloon Tong, Hong Kong SAR, China’ and ‘Department of Sport, Physical Education and Health, Hong Kong Baptist University, Kowloon Tong, Hong Kong SAR, China’. These affiliations have now been removed for author Yannis Pitsiladis.

Affiliation ‘Human Telemetrics Ltd., London, United Kingdom’ was erroneously omitted for author Yannis Pitsiladis. The conflict of interest statement has been corrected to:

“Authors WS and AK were employed by Tirol Kliniken GmbH Innsbruck. Authors YP and PV were employed by Human Telemetrics Ltd. The remaining authors declare that the research was conducted in the absence of any commercial or financial relationships that could be construed as a potential conflict of interest. The author(s) declared that they were an editorial board member of Frontiers, at the time of submission. This had no impact on the peer review process and the final decision.”

The original article has been updated.

