# Correction: Thermoregulatory responses in elite cross-country skiers during international competitions and training

**DOI:** 10.3389/fphys.2026.1800154

**Published:** 2026-02-11

**Authors:** Frontiers Production Office

**Affiliations:** Frontiers Media SA, Lausanne, Switzerland

**Keywords:** cold, competition, core temperature, cross-country skiing, skin temperature, thermoregulation

The conflict of interest statement was erroneously given as [Authors WS and AK were employed by Tirol Kliniken GmbH Innsbruck. Authors YP and PV were employed by Human Telemetrics Ltd. The remaining authors declare that the research was conducted in the absence of any commercial or financial relationships that could be construed as a potential conflict of interest. The author(s) declared that they were an editorial board member of Frontiers, at the time of submission. This had no impact on the peer review process and the final decision.].

The correct conflict of interest statement is [Authors WS and AK were employed by Tirol Kliniken GmbH Innsbruck. Author YP is the founder of Human Telemetrics Ltd. Author PV was employed by Human Telemetrics Ltd. The remaining authors declare that the research was conducted in the absence of any commercial or financial relationships that could be construed as a potential conflict of interest. The author(s) declared that they were an editorial board member of Frontiers, at the time of submission. This had no impact on the peer review process and the final decision.].

The original article has been updated.

